# New Fuzzy Support Vector Machine for the Class Imbalance Problem in Medical Datasets Classification

**DOI:** 10.1155/2014/536434

**Published:** 2014-03-23

**Authors:** Xiaoqing Gu, Tongguang Ni, Hongyuan Wang

**Affiliations:** School of Information Science and Engineering, Changzhou University, Changzhou 213164, China

## Abstract

In medical datasets classification, support vector machine (SVM) is considered to be one of the most successful methods. However, most of the real-world medical datasets usually contain some outliers/noise and data often have class imbalance problems. In this paper, a fuzzy support machine (FSVM) for the class imbalance problem (called FSVM-CIP) is presented, which can be seen as a modified class of FSVM by extending manifold regularization and assigning two misclassification costs for two classes. The proposed FSVM-CIP can be used to handle the class imbalance problem in the presence of outliers/noise, and enhance the locality maximum margin. Five real-world medical datasets, breast, heart, hepatitis, BUPA liver, and pima diabetes, from the UCI medical database are employed to illustrate the method presented in this paper. Experimental results on these datasets show the outperformed or comparable effectiveness of FSVM-CIP.

## 1. Introduction

Computer techniques such as machine learning and pattern recognition have been widely adopted by modern medicine. One reason is that an enormous amount of data has to be gathered and analyzed which is very hard or even impossible without making use of computer techniques. The other reason is that computer techniques have led toward digital analysis of pathological diagnosis, automatic classification differentiating, and detecting diseases. In some cases, an early symptom of some diseases is lighter and gives no obvious pointer to a possible diagnosis; moreover, many symptoms look very similar to each other, though they are caused by different diseases. So it may be difficult even for experienced doctors to make correct diagnosis. Therefore, an automatic classification system can help doctor diagnose accurately, assess disorders remotely and evaluate the treatment process [1].

In recent years, researchers have proposed a lot of approaches for medicine classification, such as neural network, Bayesian network, and support vector machine (SVM). Among them SVM is considered to be one of the most successful ones [2]. For example, to improve time and accuracy in differentiating diffuse interstitial lung disease for computer-aided quantification, a hierarchical SVM is introduced which shows promise for various real-time and online image-based classification applications in clinical fields [3]. SVM as a classifier is used for liver disorders and its correct classification rate is highly successful compared to the other results attained [4]. A two-stage approach is proposed for medical datasets classification, in which the artificial bee colony algorithm is used for feature selection and SVM is used for classification [5].

The support vector machine (SVM) proposed by Vapnik [6, 7] is a novel approach for solving pattern recognition problems. SVM maps the sample points into a high-dimensional feature space to seek for an optimal separating hyperplane through maximizing the margin between two classes. In addition, SVM is a quadratic programming (QP) problem that assures that its solution is obtained once it is the global unique solution, and the sparsity of solution assures better generalization. However, most of the real-world medical datasets usually contain some outliers and noisy examples. The classical SVM is very sensitive to outliers/noise. To solve this problem, fuzzy support vector machine (FSVM) [8] is proposed, in which each sample is given a fuzzy membership that denotes the attitude of the corresponding point toward one class. The membership represents how important the sample is to the decision surface.

Nevertheless, many medical datasets are composed of “normal” samples with only a small percentage of “abnormal” ones, which leads to the so-called class imbalance problems. FSM does not take into consideration the class distribution and can be sensitive to the class imbalance problem. As a result, the hyperplane of FSVM can be skewed towards the minority class, and this skewness can degrade the performance of FSVM with respect to the minority class. To tackle this problem, Veropoulos et al. [9] have proposed a method called different error costs (DEC), where the SVM objective function has been modified to assign two different misclassification cost values. It is noticed that One-Class Classification [10, 11] is sometimes used in novelty detection, and it only uses the normal training data. However, in many real medical datasets, abnormal examples exist, although they are very few. Furthermore, in classification tasks, the scatter matrix can play an important role when incorporated with local intrinsic geometry structures of samples [12]. Some methods have been recently proposed to incorporate the structure of the data distribution into SVM. A linear manifold learning method named locality preserving projection (LPP) is proposed in [13, 14], which aims at preserving the local manifold structure of the samples space. Although LPP considers enhancing the local data compactness with each manifold, it does not separate manifolds with different class labels.

In this paper, we propose a new FSVM method for the class imbalance problem (FSVM-CIP) which can be used to address both the problem of class imbalance and outliers/noise. FSVM-CIP not only considers the fuzziness of each training sample but also extends manifold regularization and maximizes the localized relative margin. It takes the positive samples and negative samples into consideration with different misclassification costs according to their unbalanced distributions. We systematically evaluated the FSVM-CIP on five real-world medical datasets and compared its performance with four different SVM methods for classification. The results showed that the proposed method can improve the classification accuracy and handle the classification problems with outliers/noise and imbalanced datasets more effectively.

The rest of this paper is organized as follows.  Section 2 briefly reviews the related works.  Section 3 presents the details of FSVM-CIP in the linear case.  Section 4 presents FSVM-CIP in the nonlinear case in detail. The experimental results on five medical datasets are reported in Section 5, and some concluding remarks are given in Section 6.

## 2. Related Works

### 2.1. Fuzzy Support Vector Machines (FSVMs)

In traditional SVM, all the data points are considered with equal importance and assigned the same penal parameter in its objective function. However, in many real-world classification applications, some sample points, such as the outliers or noises, may not be exactly assigned to one of these two classes, and each sample point does not have the same meaning to the decision surface. To solve this problem, the theory of fuzzy support vector machine was originally proposed in [8]. Fuzzy membership to each sample point is introduced such that different sample points can make different contributions to the construction of decision surface.

Suppose the training samples are

(1) $$\begin{array}{c} S=\left\{\left(x_{i},y_{i},s_{i}\right),\;\;i=1,\ldots ,N\right\}, \end{array}$$

where **x**_*i*_ ∈ **R** ^*n*^ is the *n*-dimension sample point, *y*_*i*_ ∈ {−1, +1} represents its class label, and *s*_*i*_ (*i* = 1,…, *N*) is a fuzzy membership which satisfies *σ* ≤ *s*_*i*_ ≤ 1 with a sufficiently small constant *σ* > 0. The quadratic optimization problem for classification is considered as follows:

(2) min⁡w,s,ξ 12wTw+C∑i−1lsiξis.t. yi(wTxi+b)≥1−ξi, ξi≥0,  i=1,…,l,

where **w** is a normal vector of the separating hyperplane, *b* is a bias term, and *C* is a parameter which has to be determined beforehand to control the tradeoff between the classification margin and the cost of misclassification error. Since *s*_*i*_ is the attitude of the corresponding point **x**_*i*_ towards one class and the slack variables *ξ*_*i*_ are a measure of error, then the term *s*_*i*_ *ξ*_*i*_ can be considered a measure of error with different weights. It is noted that the bigger the *s*_*i*_ is, the more importantly the corresponding point is treated; the smaller the *s*_*i*_ is, the less importantly the corresponding point is treated; thus, different input points can make different contributions to the learning of decision surface. Therefore, FSVM can find a more robust hyperplane by maximizing the margin by letting some misclassification of less important points.

In order to solve the FSM optimal problem, (2) is transformed into the following dual problem by introducing Lagrangian multipliers *α*_*i*_:

(3) max⁡α ∑i=1Nαi−12∑i=1N∑j=1Nαiαjyiyjxixjs.t.   ∑i=1Nyiαi=0, 0≤αi≤siC,  i=1,…,N.

Compared with the standard SVM, the above statement only has a little difference, which is the upper bound of the values of *α*_*i*_. By solving this dual problem in (3) for optimal *α*_*i*_, **w** and *b* can be recovered in the same way as in the standard SVM.

### 2.2. Locality Preserving Projections (LPP)

Locality preserving projection (LPP) [13, 14] is a linear dimensionality reduction algorithm by feature extraction or projection. It builds an adjacency graph incorporating neighborhood information of the data set using the Laplacian graph and then computes a transformation matrix which maps the data points into a subspace. This linear transformation optimally preserves local neighborhood information in a certain sense. The representation map generated by this method can be viewed as a linear discrete approximation to a continuous map that naturally arises from the geometry of the manifold.

For a set *X* = {**x**_*i*_} (*i* ∈ [1, *N*]), let *N*_*k*_(**x**_*i*_) denote *k* nearest neighbors of node *i*, and let *G* denote the adjacency graph of dataset *X*. Here, the *i*th node corresponds to the data point *x*_*i*_ and nodes *i* and *j* are connected by an edge if node *i* is among the *k* nearest neighbors of node *j* or if node *j* is among the *k* nearest neighbors of node *i*; that is, **x**_*i*_ ∈ *N*_*k*_(**x**_*j*_) or **x**_*j*_ ∈ *N*_*k*_(**x**_*i*_). The adjacency graph *G* can be weighed as follows:

(4) Wij={exp⁡(−||xi−xj||2t)if  xi∈Nk(xj)or  xj∈Nk(xi)0otherwise,

where exp⁡(−||**x**_*i*_−**x**_*j*_||^2^/*t*) is called the heart kernel function and *t* is a constant. ||**x**_*i*_ − **x**_*j*_|| is the Euclidean distance in **R** ^*n*^ between point *i* and point *j*. LPP tries to find the transformation vector **w** ∈ **R** ^*n*^ by minimizing the following objective function:

(5) min⁡w≠0   wTXLXTws.t. wTXDXTw=1,

where **D** is a diagonal matrix whose entries are column sum of **W** and *D*_*ii*_ = ∑_*j*_ *W*_*ij*_ normalizes each weight. **L** = **D** − **W** is the Laplacian matrix. The transformation vector **w** in the objective function in (5) is given by the minimum eigenvalue solution to the generalized eigenvalue problem. LPP preserves the intrinsic geometry and local structure of the data by minimizing the objective function.

## 3. FSVM for the Class Imbalance Problem in the Linear Case

In this section, we first define the local within-class preserving scatter matrix in the linear case. Secondly, the optimization problem formulation of FSVM-CIP in the linear case is given. Moreover, the fuzzy membership functions for linear FSVM-CIP are defined. Finally, the algorithm of linear FSVM-CIP is summarized.

### 3.1. The Local within-Class Preserving Scatter Matrix in the Linear Case

Following the idea of [15], we build the nearest within-class neighbor graph to model intrinsic geometry and local structure of the data. The graph preserves local neighborhood information in a certain sense and it can be viewed as a linear discrete approximation to a continuous map that naturally arises from the geometry of the manifold.

Considering the fact that we have a binary classification problem, one class denoted as *C*_1_ contains sample points **x**_*i*_ with *y*_*i*_ = 1 and the other class denoted as *C*_2_ contains sample points **x**_*i*_ with *y*_*i*_ = −1. Set |*C*_1_ | = *m*_1_ and |*C*_2_ | = *N* − *m*_1_, and the total number of sample points is *N*.

**Definition 1**

For each data **x**_*i*_, suppose its *k* nearest within-class neighbors set *N*_*k*_(**x**_*i*_) and an edge is put between **x**_*j*_ and its neighbors. The corresponding weight matrix *W*_*ij*_ is

(6) Wij={1Diiexp⁡(−||xi−xj||2t)if  xi∈Nk(xj) or  xj∈Nk(xi),yi=yj0otherwise,

where *D*_*ii*_ = ∑_*j*_ *W*_*ij*_ normalizes each weight.

**Definition 2**

The local within-class preserving scatter matrix

(7) Slw=∑k=12 ∑xi∈Ck(xi−∑xi∈N(xj)  or  xj∈N(xi)Wijxj)×(xi−∑xi∈N(xj)  or  xj∈N(xi)Wijxj)T=∑k=12X(k)(I(k)−W(k))T(I(k)−W(k))X(k)T,

where **I** ^(*k*)^ is an *N*_*k*_ × *N*_*k*_ diagonal matrix. In this case, the obtained nearest within-class neighbor graph attempts to preserve the local structure of the data set and (**I** ^(*k*)^ − **W** ^(*k*)^)^*T*^(**I** ^(*k*)^ − **W** ^(*k*)^) preserves locality of nearby points with same class label in the embedding space during the unfolding process of nonlinear structures [15]. In fact, a heavy penalty is applied to the objective function through the weight *W*_*ij*_ if the neighboring data **x**_*i*_ and **x**_*j*_ are mapped far apart. Hence, the minimization criterion is an attempt to ensure points *y*_*i*_ and *y*_*j*_ close to each other as well as **x**_*i*_ and **x**_*j*_ being close.

It is worthwhile to note that the local within-class scatter matrix **S**_*lw*_ is symmetric and positive semidefinite. **S**_*lw*_ looks similar to the within-class scatter matrix **S**_*w*_ [16, 17] and the Laplacian matrix **L** in LPP. However, **S**_*lw*_ reflects the intrinsic geometry and local structure of the data, and **S**_*w*_ only considers the mean value of samples in different classes. **S**_*lw*_ carries the class label information and discriminating information but **L** only considers the information of nearest neighbors for each data point in the input space, without considering the class labels.

### 3.2. FSVM-CIP in the Linear Case

**Figure 1:**
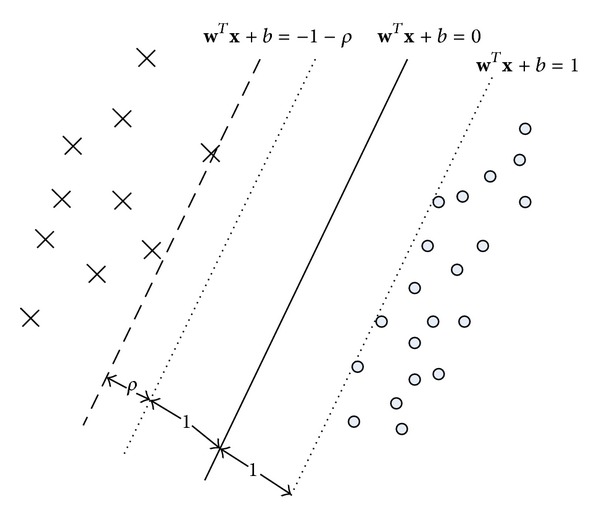
The hyperplanes of linear FSVM-CIP.

To tackle the imbalance classification problem with noise and outliers, we integrate FSVM, the ideas of imbalance classification problem, and the local within-class preserving scatter. On one hand, as shown in Figure 1, the linear classifier presented by the hyperplane is (**w** ^*T*^ **x** + *b* = 0) and defines a field for majority-class examples (**w** ^*T*^ **x** + *b* > 1 − *ξ*) and another field for minority-class examples (**w** ^*T*^ **x** + *b* > −(1 + *ρ* − *ξ*)) which is used to weaken the skewness towards the minority class and enhance the locality maximum margin. On the other hand, by assigning a higher misclassification cost for the minority class examples than the majority class examples, the effect of class imbalance could be reduced. In addition, to minimize the amount of misclassifications, the local within-class scatter matrix **S**_*lw*_ is used to preserve intrinsic geometry and local structure of the data.

Due to this, we define the primal problem of FSVM-CIP as follows:

(8) min⁡w,b,ρ,ξ 12wTw−vρ  +1v1m1∑i=1m1μiξi+1v2m2∑j=m1+1Nμjξj+η2wTSlwws.t. wTxi+b≥1−ξi, i=1,…,m1−(wTxj+b)≥1+ρ−ξj, j=m1+1,…,Nξk≥0, k=1,…,N,  ρ≥0,

where *m*_1_, *m*_2_ denote the number of positive (normal class or majority class) and negative (abnormal class or minority class) training points, and *m*_2_ = *N* − *m*_1_. *ρ* is a nonnegative number, and *ρ* + 1 is the margin between the hyperplane and the minority class examples. *η* is a nonnegative regulation constant which is the tradeoff between the local within-class scatter and the margin. Variables *v*_1_, *v*_2_ are positive penalty parameters, which tune penalty cost of the training error for positive and negative training data, respectively. *ξ*_*i*_, *ξ*_*j*_ ≥ 0 are the slack variables, and *μ*_*i*_, *μ*_*j*_ are fuzzy memberships for two-class examples.

Obviously, **w** ^*T*^ **S**_*lw*_ **w** provides prior geometrical information into the penalty terms based on manifold regularization. Minimizing **w** ^*T*^ **S**_*lw*_ **w** means that close data originally in the same class in the input space are likely to be close in the output place. Therefore, **w** ^*T*^ **S**_*lw*_ **w** aims to preserve the local information of the manifold structure.

It is noted that, in FSVM-CIP, we assign different fuzzy membership values for training examples to reflect their different classes of importance. We also showed that it is similar to assign different misclassification costs *μ*_*i*_/*v*_1_ *m*_1_(*μ*_*j*_/*v*_2_ *m*_2_) for different training examples. In order to reduce the effect of class imbalance, we can assign higher membership values *μ*_*j*_ or lower parameter *v*_2_ for the minority class examples, while we assign lower membership values *μ*_*i*_ or higher *v*_1_ for the majority class. That is, our proposed method would not tend to skew the separating hyperplane towards the minority class examples as the minority class examples are now assigned with a higher misclassification cost. By means of setting *μ*_*i*_/*v*_1_ *m*_1_(*μ*_*j*_/*v*_2_ *m*_2_) and extending manifold regularization, the learned optimal separating hyperplane enhances the relative maximum margin and FSVM-CIP will be less sensitive to imbalanced class problems.

Then, we transform this problem into its corresponding dual problem as follows.

The primal Lagrangian is

(9) L(w,b,ρ,ξ,α,γ,s) =12wTw−νρ+1v1m1∑i=1m1μiξi+1v2m2∑j=m1+1Nμjξj  +η2wTSlww−∑i=1m1αi(wTxi+b−1+ξi)  +∑j=m1+1Nαj(wTxj+b+1+ρ−ξj)−∑i=1Nγiξi−sρ,

with Lagrangian multipliers *α*_*i*_ ≥ 0, *γ*_*i*_ ≥ 0, and *s* ≥ 0. The derivatives of *L*(**w**, *b*, *ρ*, **ξ**, **α**, **γ**, *s*) with respect to the primal variables using the Karush-Kuhn-Tucker (KKT) conditions should vanish. Consider

(10) $$\begin{array}{c} \frac{\partial L}{\partial b}=\overset{N}{\underset{i=1}{\sum }}\alpha_{i}y_{i}=0, \end{array}$$

(11) $$\begin{array}{c} \frac{\partial L}{\partial w}=Iw+\eta S_{lw}w-\overset{N}{\underset{i=1}{\sum }}\alpha_{i}y_{i}x_{i}=0, \end{array}$$

(12) $$\begin{array}{c} \frac{\partial L}{\partial \rho }=-\nu +\overset{N}{\underset{j=m_{1}+1}{\sum }}\alpha_{j}-s=0, \end{array}$$

(13) $$\begin{array}{c} \frac{\partial L}{\partial \xi_{i}}=\frac{\mu_{i}}{v_{1}m_{1}}-\alpha_{i}-\gamma_{i}=0,\;i=1,\ldots ,m_{1}, \end{array}$$

(14) $$\begin{array}{c} \frac{\partial L}{\partial \xi_{j}}=\frac{\mu_{j}}{v_{2}m_{2}}-\alpha_{j}-\gamma_{j}=0,\;j=m_{1}+1,\ldots ,N, \end{array}$$

where **I** is an *N*-dimensional vector of ones, and **I** = [1,…,1]^*T*^. We have **w** = (**I** + *η* **S**_*lw*_)^−1^∑_*i*=1_^*N*^ *α*_*i*_ *y*_*i*_ **x**_*i*_.

Substituting (10)–(14) into (9), we obtain the dual form of the optimization problem:

(15) min⁡α   12αTHαs.t. ∑i=+1m1αi=v∑j=m1+1Nαj=v0≤αi≤μiv1m1, i=1,…,m10≤αj≤μjv2m2,   j=m1+1,…,N,

where **H** is a matrix with entry *H*_*ij*_ = *y*_*i*_ *y*_*j*_ **x**_*i*_^*T*^(**I** + *η* **S**_*lw*_)^−1^ **x**_*j*_, and vectors **α** = [*α*_1_,…,*α*_*N*_]^*T*^.

Equation (15) is a typical convex quadratic programming problem which is easy to be numerically solved. Suppose α* = [*α*_1_*,…, *α*_N_*] can be used to solve the above optimization problem, and then the optimal weight vector is

(16) $$\begin{array}{c} w^{*}={\left(I+\eta S_{lw}\right)}^{-1}\overset{N}{\underset{i=1}{\sum }}\alpha_{i}^{*}y_{i}x_{i}. \end{array}$$

Denote a training sample **x**_*i*_ (1 ≤ *i* ≤ *N*) called a support vector (SV) if the corresponding Lagrange multiplier *α*_*i*_ > 0. Denote the SV sets as SV_1_ = {**x**_*i*_ | 0 < *α*_*i*_ ≤ *μ*_*i*_/*v*_1_ *m*_1_, 1 ≤ *i* ≤ *m*_1_} and SV_2_ = {**x**_*j*_ | 0 < *α*_*j*_ ≤ *μ*_*j*_/*v*_2_ *m*_2_, 1 + *m*_1_ ≤ *j* ≤ *N*} while *s* ^+^ and *s* ^−^ denote the number of SVs in SV_1_ and SV_2_, respectively. According to KKT condition, (15) becomes equations for the input data in SV_1_ and SV_2_, respectively, with slack variables *ξ*_*i*_ and *ξ*_*j*_ being 0. Thus, the optimal thresholds b* and ρ* can be calculated. However, from the numerical perspective, it is better to take the mean value of b* and ρ* resulting from all such data. Therefore, the optimal thresholds b* and ρ* are computed by the following formula:

(17) $$\begin{array}{c} b^{*}=1-\frac{1}{s^{+}}\underset{x_{i}\in \text{S}{\text{V}}_{1}}{\sum }{\left(w^{*}\right)}^{T}x_{i}, \end{array}$$

(18) $$\begin{array}{c} \rho^{*}=-\frac{1}{s^{+}}\underset{x_{i}\in \text{S}{\text{V}}_{1}}{\sum }{\left(w^{*}\right)}^{T}x_{i}+\frac{1}{s^{-}}\underset{x_{j}\in \text{S}{\text{V}}_{2}}{\sum }{\left(w^{*}\right)}^{T}x_{j}. \end{array}$$

As a result, the corresponding decision function of the linear FSVM-CIP will be

(19) f(x)=sgn⁡(wTx+b∗)=sgn⁡(∑i=1Nαi∗yi(xiT(I+ηSlw)−1x)+b∗).

Note that, to deal with the small sample size problem, (**I** + *η* **S**_*lw*_) is regularized by adding a scale multiple *η* of the identity matrix **S**_*lw*_ with **I** before any inversion takes place. Hence, (**I** + *η* **S**_*lw*_) is always nonsingular, and the inverse of (**I** + *η* **S**_*lw*_) exists.

Following the terminology in [18], a training sample **x**_*i*_ (1 ≤ *i* ≤ *N*) is called a margin error (ME) if the corresponding slack variable **ξ**_*i*_ > 0. We give the following theorem for parameter selection later.

**Theorem 3**

Let *m* ^+^ and *m* ^−^ denote the number of MEs in the positive and negative classes; *s* ^+^ and *s* ^−^ denote the number of SVs in the positive and negative classes, respectively. Then one has

(20) $$\begin{array}{c} \bar{\mu_{m}^{+}}m^{+}\leq vv_{1}m_{1}\leq \bar{\mu_{s}^{+}}s^{+}, \end{array}$$

(21) $$\begin{array}{c} \bar{\mu_{m}^{-}}m^{-}\leq vv_{2}m_{2}\leq \bar{\mu_{s}^{-}}s^{-}, \end{array}$$

where $\bar{\mu_{m}^{+}}$ and $\bar{\mu_{m}^{-}}$ denote the mean fuzzy membership of MEs in the positive and negative classes; $\bar{\mu_{s}^{+}}$ and $\bar{\mu_{s}^{-}}$ denote the mean fuzzy membership of SVs in the positive and negative classes, respectively.

A proof of the above theorem can be found in Appendix.

### 3.3. Fuzzy Membership Functions in the Linear Case

In FSVM, the fuzzy membership is used to reduce the effects of outliers or noises and different fuzzy membership functions have different influences on the fuzzy algorithm. Basically, the rule to assign proper membership values to data points can depend on the relative importance of date points to their own classes. In this paper, we consider two fuzzy membership functions given in [19].

Given the sequence of training points, denote the mean of positive class and negative class as ${\overset{-}{x}}_{+}$ and ${\overset{-}{x}}_{-}$.

**Definition 4**

The *μ*_*lin*⁡_ is called the linear fuzzy membership and *μ*_*lin*⁡_ can be defined as

(22) μlin⁡={1−||xi−x−+||(max⁡j(||xj−x−+||)+δ)if  yi=11−||xi−x−−||(max⁡j(||xj−x−−||)+δ)if  yi=−1,

where *δ* is a small positive value, which is used to avoid *μ*_*lin*⁡_ becoming zero. ||·|| is the Euclidean distance.

**Definition 5**

The *μ*_exp⁡_ is called the exponential fuzzy membership and *μ*_exp⁡_ can be defined as

(23) μexp⁡={21+exp⁡(λ||xi−x−+||)if  yi=121+exp⁡(λ||xi−x−−||)if  yi=−1,

where parameter *λ* ∈ [0,1] determines the steepness of the decay.

### 3.4. Solution

Based on the above, we can state the approach of proposed FSVM-CIP in the linear case as Algorithm 1.

## 4. FSVM for the Class Imbalance Problem in the Nonlinear Case

In this section, we extend the local within-class preserving scatter matrix and FSVM-CIP into feature space. Moreover, the fuzzy membership functions in feature space are defined. Finally, the algorithm of kernel FSVM-CIP is summarized.

### 4.1. Kernel Extension

In order to handle nonlinear classification, the kernelization trick [20] is used to map the *n*-dimensional date points into an arbitrary reproducing kernel Hilbert space (RKHS) [21] via a mapping function *ϕ* : **R** ^*n*^ ↦ **H**; that is, **x**_**i**_ ↦ *ϕ*(**x**_**i**_). Then a linear hyperplane *f*(**v**) = **α** ^*T*^ *ϕ*(**v**) + *b* in feature space **H** would correspond to a nonlinear hyperplane in the original space **R** ^*n*^ where **α**, *ϕ*(**v**) ∈ **H**, **v** ∈ **R** ^*n*^, and *b* ∈ **R**.

Let *ϕ*(**X**) denote the date matrices in feature space **H**, *ϕ*(**X**) = [*ϕ*(**x**_1_), *ϕ*(**x**_2_),…, *ϕ*(**x**_*n*_)]; then the kernel function **K** is a matrix with entry *K*_*ij*_ = *K*(**x**_*i*_, **x**_*j*_) = *ϕ*(**x**_*i*_)^*T*^ *ϕ*(**x**_*j*_).

Here the kernel local within-class scatter matrix **S**_*lw*_^*ϕ*^ in feature space is

(24) Slwϕ=∑k=12∑i=1Nk(ϕ(xi)−∑j=1NkWijϕkϕ(xj))×(ϕ(xi)−∑j=1NkWijϕkϕ(xj))T=K(1)(I(1)−Wϕ(1))T(I(1)−Wϕ(1))K(1)T +K(2)(I(2)−Wϕ(2))T(I(2)−Wϕ(2))K(2)T,

where **I** ^(1)^, **I** ^(2)^ are *N*_1_-order, *N*_2_-order identity matrixes, respectively. Based on the above notations, **K** ^(1)^, **K** ^(2)^ are *N* × *m*_1_,  *N* × (*N* − *m*_1_) matrixes, respectively; thus **K** = [**K** ^(1)^, **K** ^(2)^].

The weight matrixes **W** ^*ϕ*(1)^ and **W** ^*ϕ*(2)^ are the nonlinear version of **W** ^(1)^  and **W** ^(2)^, respectively. **W** ^*ϕ*(1)^and **W** ^*ϕ*(2)^ could be built by *W*_*ij*_^*ϕ*^, and the nonlinear version of *W*_*ij*_^*ϕ*^ is

(25) Wijϕ={1Diiϕexp⁡(−(Kii+Kjj−2Kij)t)if  xi∈Nk(xj) or  xj∈Nk(xi),yi=yj0otherwise,

where *D*_*ii*_^*ϕ*^ = ∑_*j*_ *W*_*ij*_^*ϕ*^ is a normalizer.

Thus, the kernel FSVM-CIP can be easily achieved by solving the following quadratic problem:

(26) min⁡w,b,ρ,ξ 12wTw−vρ+1v1m1∑i=1m1μiξi+1v2m2∑j=m1+1Nμjξj+η2wTSlwϕws.t.   wTϕ(xi)+b≥1−ξi, i=1,…,m1wTϕ(xj)+b≥1+ρ−ξj, j=m1+1,…,Nξk≥0, k=1,…,N,  ρ≥0.

Like its linear counterpart, the solution to this optimization problem can be easily found using Lagrange multipliers. By using the representer theorem, **w** can be given by **w** = ∑_*i*=1_^*N*^ *β*_*i*_ *ϕ*(**x**_*i*_). We obtain the dual form of the optimization problem:

(27) min⁡α 12αTMαs.t. ∑i=1m1αi=v∑j=m1+1Nαj=v0≤αi≤μiv1m1, i=1,…,m10≤αj≤μjv2m2, j=m1+1,…,N,

where **M** = **Y** **K** ^*T*^ **Q** ^−1^ **K** **Y** and **Q** = **K** + *η* **K** ^(1)^(**I** ^(1)^ − **W** ^*ϕ*^ ^(1)^)^*T*^(**I** ^(1)^ − **W** ^*ϕ*^ ^(1)^)**K** ^(1)*T*^ + *η* **K** ^(2)^(**I** ^(2)^ − **W** ^*ϕ*^ ^(2)^)^*T*^(**I** ^(2)^ − **W** ^*ϕ*^ ^(2)^)**K** ^(2)*T*^. Vectors **α** = [*α*_1_,…, *α*_*N*_]^*T*^, and *Y* = diag⁡(*y*_1_, *y*_2_,…, *y*_*n*_) is a diagonal matrix.

Equation (27) is a typical convex quadratic programming problem which is easy to be numerically solved. Suppose α* = [*α*_1_*,…, *α*_N_*]^*T*^ can be used to solve the above optimization problem; then the optimal weight vector β* = **Q** ^−1^ **K** **Y** α*. Therefore, the optimal thresholds b* and ρ* are computed by the following formula:

(28) $$\begin{array}{c} b^{*}=1-\frac{1}{s^{+}}\underset{x_{i}\in \text{s}{\text{v}}_{1}}{\sum }\overset{N}{\underset{j=1}{\sum }}\beta_{j_{}}^{*}y_{j}K(x_{i},x_{j}), \end{array}$$

(29) ρ∗=−1s+∑xi∈SV1∑j=1Nβj∗yjK(xi,xj)+1s−∑xi∈SV2∑j=1Nβj∗yjK(xi,xj).

Finally, a more robust decision function of kernel FSVM-CIP will be

(30) $$\begin{array}{c} f\left(x\right)=\mathrm{sgn}\left(\overset{N}{\underset{i=1}{\sum }}\beta_{i}^{*}K\left(x,x_{i}\right)+b^{*}\right). \end{array}$$

**Theorem 6**

The matrix **M** in (27) is symmetric and positive semidefinite.

A proof of the above theorem can be found in Appendix.

Next, we consider fuzzy membership functions in feature space.

**Definition 7**

The *μ*_*lin*⁡_^*ϕ*^ is called the linear fuzzy membership in feature space and *μ*_*lin*⁡_^*ϕ*^ can be defined as

(31) μlin⁡ϕ={1−||ϕ(xi)−ϕ(x−+)||(max⁡j(||ϕ(xj)−ϕ(x−+)||)+δ)  if  yi=11−||ϕ(xi)−ϕ(x−−)||(max⁡j(||ϕ(xj)−ϕ(x−−)||)+δ)if  yi=−1,

where *δ* is a small positive value. ||·|| is the Euclidean distance.

**Definition 8**

The *μ*_exp⁡_^*ϕ*^ is called the exponential fuzzy membership in feature space and *μ*_exp⁡_^*ϕ*^ can be defined as

(32) μexp⁡ϕ={21+exp⁡(λ||ϕ(xi)−ϕ(x−+)||)if  yi=121+exp⁡(λ||ϕ(xi)−ϕ(x−−)||)if  yi=−1,

where parameter *λ* ∈ [0,1] determines the steepness of the decay. Consider

(33) $$\begin{array}{c} \phi \left({\overset{-}{x}}_{+}\right)=\frac{1}{m_{1}}\underset{x_{i}\in c_{1}}{\sum }\phi (x_{i}),\\ \phi \left({\overset{-}{x}}_{-}\right)=\frac{1}{N-m_{1}}\underset{x_{i}\in c_{2}}{\sum }\phi (x_{i}). \end{array}$$

Thus, the distance $||\phi (x_{i})-\phi ({\overset{-}{x}}_{+})||$ can be given by

(34) ||ϕ(xi)−ϕ(x−+)||=K(xi,xi)−2m1∑xj∈C1K(xi,xj)+1m12∑xs∈C1∑xt∈C1K(xs,xt).

Likewise, the $||\phi (x_{i})-\phi ({\overset{-}{x}}_{-})||$ can be given in a similar manner.

### 4.2. Solution

Based on the above, we can state the approach of kernel FSVM-CIP as Algorithm 2.

## 5. Experiments and Discussions

To evaluate the performance of our proposed FSVM-CIP, in this section, FSVM-CIP is evaluated compared with other related representative methods, such as standard FSVM [8], SVDD [11], FSVM for class imbalance learning (FSVM-CIL) [22], and FSVM with minimum within-class scatter (WCS-FSVM) [23]. We implement FSVM-CIP using the linear fuzzy membership and the exponential fuzzy membership, respectively, which are represented as FSVM-CIP_*lin*⁡_ and FSVM-CIP_exp⁡_. All the experiments are performed in Matlab (R2010a) on personal computer, whose configuration is as follows: CPU 2.99 GHz, 4.0 G RAM, and Microsoft Windows XP.

### 5.1. Data Preparation

In this section, we use five real-world medical datasets from the UCI repository of machine learning database [24], to demonstrate the classification performance of the method proposed in this paper. These five medical datasets are breast, heart, hepatitis, BUPA liver, and pima diabetes. It is highly likely that these real-world datasets contain some outliers and noisy examples in different amounts [22]. In each of them, the positive class consists of the data corresponding to the healthy, normal, or benign cases, while the negative class contains the data for diseased, abnormal, or malignant cases. Further details of these datasets are provided in Table 1. This contains the total number of positive data #pos, the total number of negative data #neg, the number of positive training examples *m*1, the number of negative training examples *m*2, the positive-to-negative imbalance ratio Ratio, and the data dimensionality *d*.

### 5.2. Performance Measure and Experimental Settings

We used the geometric mean of sensitivity (sensitivity = proportion of the positives correctly recognized), specificity (specificity = proportion of the negatives correctly recognized), and accuracy (accuracy = proportion of correctly classified instances) for the classifier performance evaluation in experiments, as commonly used in medical datasets classification research [7].

Like the existing SVM and FSVM algorithms, the solution is sensitive to the setting of the parameters. In order to evaluate the performance, a strategy is that a set of the parameters is given first and then the best cross-validation mean rate among the set is used to estimate the generalized accuracy. We adopt this strategy in this paper. For FSVM-CIP, the parameter *ν* is searched in {1, 5, 10, 15, …, 80}, while *v*_1_ and *v*_2_ are selected from {0.001, 0.005, 0.01, 0.05}. *η* is selected from log⁡_2_ *η* ∈ {−5, − 4.5, − 4, …, 5.5, 6}. The heat kernel parameter *t* is searched in {0.5, 1.0, 1.5, 2.0, 2.5, 3.0, 3.5, 4.0} and the neighborhood parameter *k* is searched in {3, 5, 7, 9, 11, 13, 15}. In addition, when the linear fuzzy function is used, we set *δ* = 10^−6^. When the exponential fuzzy function is used, the optimal value of *λ* is chosen from the range *λ* = {0.1, 0.2, 0.3, …, 1}.

The regularization parameter *C* for FSVM, SVDD, FSVM-CIL, and WCS-FSVM is selected from the set {0.001, 0.01, 0.1, 1, 10, 100}. In WCS-FSVM, *β* is selected from log⁡_2_ *β* ∈ {−5, − 4.5, − 4, …, 5.5, 6}. For FSVM-CIL, the fuzzy membership is based on the distance from the actual hyperplane and uses the exponential fuzzy membership *λ*. *λ* is chosen from the range *λ* = {0.1, 0.2, 0.3, …, 1}.

For the kernel-based methods, we use a Gaussian RBF kernel, that is, exp⁡(−(*u* − *v*)^*T*^(*u* − *v*)/*σ*), where *σ* is the spread of Gaussian kernel, and *σ* is searched in {*τ* ^2^/16, *τ* ^2^/8, *τ* ^2^/4, *τ* ^2^/2, *τ* ^2^, 2*τ* ^2^, 4*τ* ^2^, 8*τ* ^2^, 16*τ* ^2^}, where *τ* ^2^ is the mean norm of the training data.

For parameter selection, we conduct fivefold cross-validation in a stratified manner so that each validation set has the same positive to negative ratio as in the training set. Finally, the experiment is repeated 10 times independently of each dataset.

### 5.3. Experimental Results

FSVM-CIP method test results developed for the breast, heart, hepatitis, BUPA liver, and pima diabetes datasets are given both in the linear case and nonlinear case. Tables 2, 3, 4, 5, and 6 display the comparison results with the other methods on these five databases, respectively.

The main observations from the performance comparisons include the following.

(1) We can see that, in many real-world applications, a linear classifier seems powerless. In terms of accuracy, kernel method can improve the classification performance for all five medical datasets.

(2) We can clearly observe that the FSVM-CIP outperforms other methods on almost datasets both in the linear case and nonlinear case, which gives higher accuracy. This fortifies the fact that the locality maximum margin and the local structure information presented by local within-class preserving scatter could improve classification performance; furthermore, the method of different misclassification costs based on the number of two classes is a sensitive learning solution to overcome the imbalance problem in SVMs.

(3) It is noted that, for all the datasets considered, the classification accuracy given by the FSVM-CIP_exp⁡_ setting is higher than the FSVM-CIP_*lin*⁡_ setting. Therefore, we can state that FSVM-CIP_exp⁡_ setting with the appropriate selection of *λ* value would be an effective choice applied to any medical dataset. In other words, when dealing with medical datasets classification, the performance of the exponential fuzzy membership is better than linear fuzzy membership in FSVM-CIP.

(4) For breast and heart datasets, the class imbalance is not obviously shaped; WCS-FSVM yielded standard FSVM, SVDD, and FSVM-CIL. We can say that the performance can indeed be improved when the structure of the data is taken into consideration. For the other three datasets, the class imbalance strikingly improved, the results given by standard FSVM and WCS-FSVM for datasets are biased towards the majority class represented as lower specificity and lower accuracy. These results justify the fact that these two methods are sensitive to the class imbalance problem. Meanwhile, SVDD and FSVM-CIL yielded standard FSVM and WCS-FSVM. BY assigning different misclassification costs for the minority class and majority class, the effect of class imbalance could be reduced.

### 5.4. Parameter Selection for Kernel FSVM-CIP_exp⁡_

**Figure 2:**
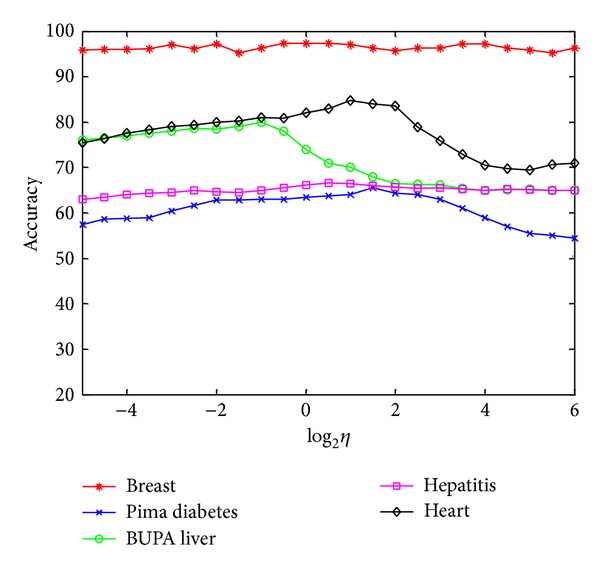
The effect of the parameter *η* on kernel FSVM-CIP_exp⁡_.

The parameter *η* > 0 is an essential parameter in our proposed method which controls the tradeoff between the local within-class scatter and the margin.  Figure 2 shows the impact of parameter *η* on the classification accuracy of FSVM-CIP_exp⁡_ in kernel case with each value of *η* selected from log⁡_2_ *η* ∈ {−5, − 4.5, − 4, …, 5.5, 6}. It can be seen that the best accuracy is obtained for all the datasets and therefore *η* is searched in a reasonable range.

**Figure 3:**
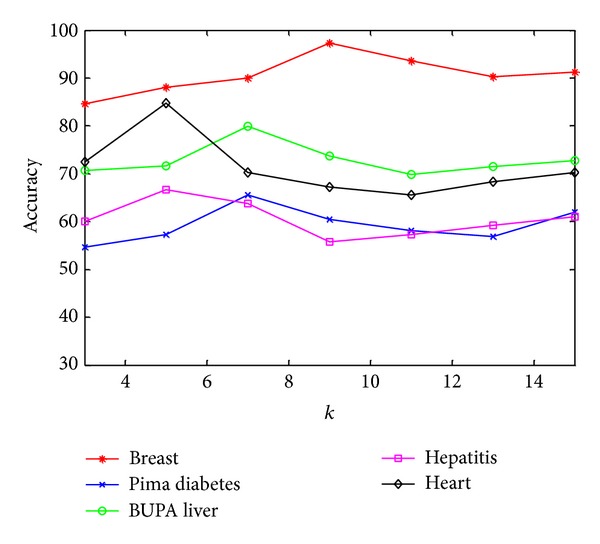
The effect of the parameter *k* on kernel FSVM-CIP_exp⁡_.

Compared with standard FSVM, the additional neighbor parameter *k* is employed in FSVM-CIP. To evaluate the influence of this parameter on the performance, the classification accuracy of kernel FSVM-CIP_exp⁡_ for five medical databases is recorded for each value of *k* in {3, 5, 7, 9, 11, 13, 15}.  Figure 3 shows the results. It can be seen that the classification accuracy is not high when *k* value is small and, by increasing *k*, the classification accuracy increases; however, if *k* continues to increase, the classification accuracy begins to drop severely down. It is because, when *k* is too small, the number of nearest neighbors is sparse; when *k* is too large, the number of nearest neighbors is excessive, so to preserve so much local relation may be inappropriate.

## 6. Conclusion

Computer tools have improved the medical practice implementation to a greater extent. Although computer tools cannot replace the doctors, they can make their work easier and more effective. In this paper, a new fuzzy support machine called FSVM-CIP, used for medical datasets classification, is proposed. The proposed method is based on local within-class preserving scatter and assigned two misclassification costs in the SVM objective function, which is for learning from imbalance datasets in the presence of outliers/noise and enhancing the locality maximum margin. Experiments were performed on several UCI medical datasets with a comparison of the proposed method with several other related methods such as standard FSVM, SVDD, FSVM-CIL, and WCS-FSVM. Obtained results show that the performance of the proposed method is highly successful compared to other results attained and seems very promising. Finally, we can recommend that FSVM-CIP_exp⁡_ which uses the exponential fuzzy membership would be an effective choice for medical datasets classification applications. In future work, we intend to perform investigations to large-scale classification problems.

## Figures and Tables

**Algorithm 1:**
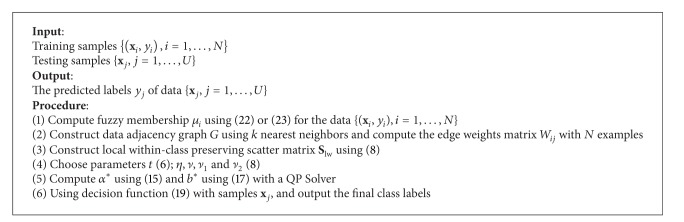
FSVM-CIP in the linear case.

**Algorithm 2:**
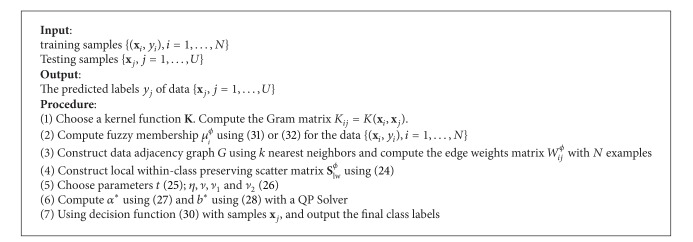
Kernel FSVM-CIP.

**Table 1:** Characteristics of the selected datasets.

Datasets	#pos	#neg	*m*1	*m*2	Ratio	*d*
Breast	458	241	240	120	2 : 1	9
Heart	120	150	80	20	4 : 1	13
Hepatitis	123	32	100	10	10 : 1	19
BUPA liver	200	145	150	10	15 : 1	6
Pima diabetes	268	500	180	10	18 : 1	8

**Table 2:** Comparison of the classification results (%) on breast dataset.

	Method	Sensitivity	Specificity	Accuracy
Linear	FSVM	95.87 ± 0.017	95.04 ± 0.043	95.58 ± 0.035
	SVDD	97.71 ± 0.065	90.90 ± 0.013	95.28 ± 0.052
	FSVM-CIL	95.87 ± 0.024	95.87 ± 0.015	95.81 ± 0.028
	WCS-FSVM	96.33 ± 0.067	95.04 ± 0.056	95.87 ± 0.047
	FSVM-CIP_lin_	96.98 ± 0.039	96.49 ± 0.022	96.76 ± 0.040
	FSVM-CIP_exp_	96.68 ± 0.011	96.69 ± 0.042	96.76 ± 0.037

Gaussian kernel	FSVM	96.33 ± 0.023	95.87 ± 0.051	96.17 ± 0.050
	SVDD	97.30 ± 0.065	91.25 ± 0.013	95.44 ± 0.052
	FSVM-CIL	96.79 ± 0.059	95.87 ± 0.042	96.46 ± 0.055
	WCS-FSVM	96.97 ± 0.030	96.69 ± 0.093	96.76 ± 0.067
	FSVM-CIP_lin_	97.25 ± 0.055	96.29 ± 0.032	97.05 ± 0.042
	FSVM-CIP_exp_	97.25 ± 0.055	97.52 ± 0.045	97.34 ± 0.033

**Table 3:** Comparison of the classification results (%) on heart dataset.

	Method	Sensitivity	Specificity	Accuracy
Linear	FSVM	87.50 ± 0.080	80.77 ± 0.069	82.35 ± 0.069
	SVDD	87.03 ± 0.021	77.69 ± 0.005	80.00 ± 0.051
	FSVM-CIL	85.00 ± 0.046	82.04 ± 0.110	82.35 ± 0.072
	WCS-FSVM	87.30 ± 0.071	81.54 ± 0.089	82.94 ± 0.088
	FSVM-CIP_lin_	85.00 ± 0.063	82.31 ± 0.083	82.84 ± 0.054
	FSVM-CIP_exp_	87.50 ± 0.025	82.31 ± 0.083	83.53 ± 0.055

Gaussian kernel	FSVM	86.70 ± 0.099	82.61 ± 0.087	83.35 ± 0.042
	SVDD	90.35 ± 0.022	80.77 ± 0.034	82.80 ± 0.070
	FSVM-CIL	87.05 ± 0.034	81.54 ± 0.067	82.94 ± 0.044
	WCS-FSVM	91.00 ± 0.076	81.73 ± 0.083	84.12 ± 0.085
	FSVM-CIP_lin_	90.00 ± 0.045	82.31 ± 0.086	84.12 ± 0.052
	FSVM-CIP_exp_	86.05 ± 0.023	83.08 ± 0.078	84.71 ± 0.066

**Table 4:** Comparison of the classification results (%) on hepatitis dataset.

	Method	Sensitivity	Specificity	Accuracy
Linear	FSVM	82.60 ± 0.053	22.73 ± 0.087	53.33 ± 0.073
	SVDD	73.91 ± 0.071	45.45 ± 0.011	60.00 ± 0.046
	FSVM-CIL	77.66 ± 0.026	45.46 ± 0.082	61.02 ± 0.070
	WCS-FSVM	79.56 ± 0.107	27.27 ± 0.062	53.33 ± 0.059
	FSVM-CIP_lin_	78.26 ± 0.046	45.46 ± 0.032	62.22 ± 0.023
	FSVM-CIP_exp_	78.26 ± 0.068	50.00 ± 0.086	64.44 ± 0.071

Gaussian kernel	FSVM	73.91 ± 0.038	31.82 ± 0.012	53.33 ± 0.025
	SVDD	82.60 ± 0.053	42.86 ± 0.025	63.64 ± 0.030
	FSVM-CIL	77.26 ± 0.041	50.00 ± 0.086	63.84 ± 0.064
	WCS-FSVM	78.26 ± 0.015	36.36 ± 0.074	57.78 ± 0.056
	FSVM-CIP_lin_	73.51 ± 0.064	54.55 ± 0.037	64.44 ± 0.058
	FSVM-CIP_exp_	73.91 ± 0.050	59.10 ± 0.011	66.67 ± 0.036

**Table 5:** Comparison of the classification results (%) on BUPA liver dataset.

	Method	Sensitivity	Specificity	Accuracy
Linear	FSVM	88.10 ± 0.008	66.42 ± 0.073	72.19 ± 0.057
	SVDD	87.27 ± 0.021	68.05 ± 0.063	72.72 ± 0.042
	FSVM-CIL	88.00 ± 0.004	67.44 ± 0.042	73.19 ± 0.015
	WCS-FSVM	84.00 ± 0.360	67.15 ± 0.068	71.66 ± 0.051
	FSVM-CIP_lin_	88.00 ± 0.004	67.88 ± 0.063	73.26 ± 0.031
	FSVM-CIP_exp_	86.00 ± 0.048	69.34 ± 0.072	73.80 ± 0.054

Gaussian kernel	FSVM	96.00 ± 0.057	66.67 ± 0.026	74.60 ± 0.038
	SVDD	95.43 ± 0.033	71.24 ± 0.050	77.23 ± 0.017
	FSVM-CIL	95.00 ± 0.045	72.59 ± 0.052	78.37 ± 0.050
	WCS-FSVM	90.08 ± 0.070	67.44 ± 0.083	73.73 ± 0.062
	FSVM-CIP_lin_	94.00 ± 0.049	74.10 ± 0.045	79.46 ± 0.048
	FSVM-CIP_exp_	94.00 ± 0.049	73.33 ± 0.084	79.92 ± 0.074

**Table 6:** Comparison of the classification results (%) on pima diabetes dataset.

	Method	Sensitivity	Specificity	Accuracy
Linear	FSVM	91.91 ± 0.022	49.98 ± 0.053	55.36 ± 0.051
	SVDD	88.65 ± 0.081	53.43 ± 0.062	58.45 ± 0.029
	FSVM-CIL	86.36 ± 0.064	55.10 ± 0.059	59.86 ± 0.060
	WCS-FSVM	87.50 ± 0.043	52.65 ± 0.024	57.96 ± 0.030
	FSVM-CIP_lin_	85.23 ± 0.021	57.76 ± 0.064	61.94 ± 0.043
	FSVM-CIP_exp_	84.09 ± 0.009	57.96 ± 0.062	61.94 ± 0.053

Gaussian kernel	FSVM	93.18 ± 0.031	51.02 ± 0.073	57.44 ± 0.053
	SVDD	91.76 ± 0.025	56.86 ± 0.052	62.57 ± 0.028
	FSVM-CIL	90.91 ± 0.047	58.78 ± 0.084	63.67 ± 0.077
	WCS-FSVM	92.05 ± 0.010	54.69 ± 0.066	60.38 ± 0.053
	FSVM-CIP_lin_	88.84 ± 0.040	61.38 ± 0.063	65.57 ± 0.063
	FSVM-CIP_exp_	88.64 ± 0.029	61.43 ± 0.074	65.57 ± 0.070

## Appendix

Proof of Theorem 3 in Section 3.2.

Proof According to the dual form of the optimization problem (15), we can derive

(A.1) $$\begin{array}{c} \overset{m_{1}}{\underset{i=1}{\sum }}\alpha_{i}=v. \end{array}$$

Likewise, according to the KKT conditions, ∑_*i*=1_^*N*^ *α*_*i*_ = *v* with *ρ* > 0 satisfy *s* = 0 by (12). According to (11), all samples with **ξ**_*i*_ > 0 satisfy *γ*_*i*_ = 0. In view of (13), this implies that *α*_*i*_ = *μ*_*i*_/*v*_1_ *m*_1_ holds for every positive ME. Summing up *α*_*i*_ over the positive MEs using (A.1), we have

(A.2) $$\begin{array}{c} \frac{\bar{\mu_{m}^{+}}m^{+}}{v_{1}m_{1}}\leq \overset{m_{1}}{\underset{i=1}{\sum }}\alpha_{i}=\nu . \end{array}$$

Furthermore, in view of (15), each SV in the positive class can control at most 1/*v*_1_ *m*_1_ to the ∑_*i*=1_^*m*_1_^ *α*_*i*_; as a result,

(A.3) $$\begin{array}{c} \overset{m_{1}}{\underset{i=1}{\sum }}\alpha_{i}\leq \frac{\bar{\mu_{s}^{+}}s^{+}}{v_{1}m_{1}}. \end{array}$$

Combining (A.2) and (A.3), inequality (20) can hold true. Likewise, inequality (21) can be proven in a similar manner.

Proof of Theorem 6 in Section 4.1.

Proof We know that **M** = **Y** **K** ^*T*^ **Q** ^−1^ **K** **Y**, and **K** is a Gram matrix, so **K** is symmetric and positive semidefinite. The transpose of the matrix **Q** is

(A.4) QT=(K+ηK(1)(I(1)−Wϕ(1))T(I(1)−Wϕ(1))K(1)T  +ηK(2)(I(2)−Wϕ(2))T(I(2)−Wϕ(2))K(2)T)T=Q.

So **Q** is a symmetric matrix and then **M** is symmetric. Set **Q** = **K** + *η* **R**, where

(A.5) R=K(1)(I(1)−Wϕ(1))T(I(1)−Wϕ(1))K(1)T  +K(2)(I(2)−Wϕ(2))T(I(2)−Wϕ(2))K(2)T.

For any nonzero vector **u** = (*u*_1_, *u*_2_,…, *u*_*N*_)^*T*^,

(A.6) uTRu=uTK(1)(I(1)−Wϕ(1))T(I(1)−Wϕ(1))K(1)Tu   +uTK(2)(I(2)−Wϕ(2))T(I(2)−Wϕ(2))K(2)Tu=ςTSlwϕς≥0,

where **ς** = ∑_*i*=1_^*N*^ *u*_*i*_ *ϕ*(**x**_*i*_). The local within-class scatter matrix **S**_*lw*_^*ϕ*^ is semidefinite, so the matrix **R** is semidefinite. That is, the matrix **Q** is semidefinite, and then **M** is semidefinite.
